# Identification of metabolic pathways and enzyme systems involved in the in vitro human hepatic metabolism of dronedarone, a potent new oral antiarrhythmic drug

**DOI:** 10.1002/prp2.44

**Published:** 2014-04-22

**Authors:** Sylvie Klieber, Catherine Arabeyre-Fabre, Patricia Moliner, Eric Marti, Martine Mandray, Robert Ngo, Céline Ollier, Priscilla Brun, Gérard Fabre

**Affiliations:** SANOFI-AVENTIS Recherche & Development Disposition, Safety and Animal Research Scientific Core Platform, Drug Disposition Domain371 Rue du Professeur Joseph Blayac, 34184 Montpellier, Cedex 4, France

**Keywords:** Antiarrhythmic drug, cytochrome P450, dronedarone metabolism, metabolite identification, monoamine oxidase

## Abstract

The in vitro metabolism of dronedarone and its major metabolites has been studied in human liver microsomes and cryopreserved hepatocytes in primary culture through the use of specific or total cytochrome P450 (CYP) and monoamine oxidase (MAO) inhibitors. The identification of the main metabolites and enzymes participating in their metabolism was also elucidated by using *rh*CYP, *rh*MAO, flavin monooxygenases (*rh*FMO) and UDP-glucuronosyltransferases (*rh*UGT) and liquid chromatography/tandem mass spectrometry (LC/MS-MS) analysis. Dronedarone was extensively metabolized in human hepatocytes with a metabolic clearance being almost completely inhibited (98 ± 2%) by 1-aminobenzotriazole. Ketoconazole also inhibited dronedarone metabolism by 89 ± 7%, demonstrating the crucial role of CYP3A in its metabolism. CYP3A isoforms mostly contributed to *N*-debutylation while hydroxylation on the butyl-benzofuran moiety was catalyzed by CYP2D6. However, hydroxylation on the dibutylamine moiety did not appear to be CYP-dependent. *N*-debutyl-dronedarone was less rapidly metabolized than dronedarone, the major metabolic pathway being catalyzed by MAO-A to form propanoic acid-dronedarone and phenol-dronedarone. Propanoic acid-dronedarone was metabolized at a similar rate to that of *N*-debutyl-dronedarone and was predominantly hydroxylated by CYP2C8 and CYP1A1. Phenol-dronedarone was extensively glucuronidated while *C*-dealkyl-dronedarone was metabolized at a slow rate. The evaluation of the systemic clearance of each metabolic process together with the identification of both the major metabolites and predominant enzyme systems and isoforms involved in the formation and subsequent metabolism of these metabolites has enhanced the overall understanding of metabolism of dronedarone in humans.

## Introduction

Atrial fibrillation (AF) is the most common type of arrhythmia in the United States; this pathology affects ∼2.5 million people. AF is a major cause of morbidity and mortality, resulting in a fivefold increased risk of stroke, an increased risk of death, and a decreased health-related quality of life (Rosamond et al. 2008). Amiodarone is one of the most frequently used drugs to maintain normal sinus rhythm (NSR); however, this agent is associated with a number of serious side effects and troublesome drug interactions, as well as complicated dosing because of a large volume of distribution and long half-life (McNamara et al. 2003).

**Figure 1:**
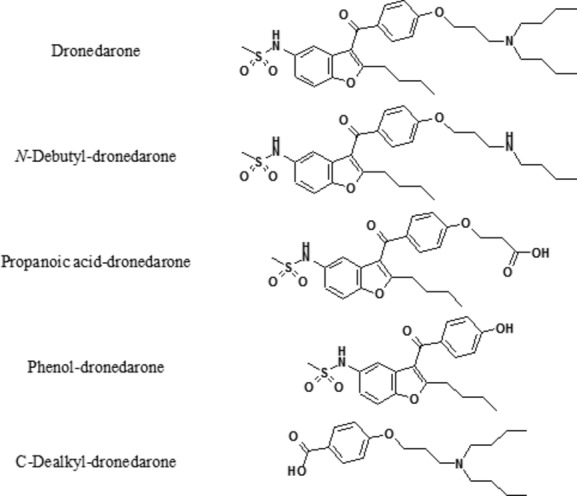
Chemical structures of dronedarone and its metabolites.

Dronedarone [also known as *N*-(2-Butyl-3-(4-(3-(dibutylamino)propoxy)benzoyl)-5-benzofuranyl)methanesulfonamide, hydrochloride] is a noniodinated benzofuran derivative (Fig. 1) (Tafreshi and Rowles 2007; Watanabe and Kimura 2008) with electrophysiological properties that are very similar to those of amiodarone; both agents belonging to all four Vaughan-Williams classes. Consequently, amiodarone-like antiarrhythmic actions, including sodium-channel blocking at rapid pacing rates (class I effect), prolonged cardiac action potentials and refractoriness (class III effect), calcium-channel antagonism (class IV effect), and noncompetitive antiadrenergic effects (class II effect), have been demonstrated with dronedarone in rat, dog, and human hearts (Watanabe and Kimura 2008). As a result of these channel-blocking effects, there is a dose-related increase in the PR and QT intervals with dronedarone doses up to 1600 mg/day (Touboul et al. 2003; Wadhani et al. 2006) and with the 400-mg twice daily dose typically used in clinical trials, the PR interval increased by 13.4 msec, and the incidence of any QTc interval >500 msec was 7.7% (Touboul et al. 2003; Wadhani et al. 2006). Dronedarone has obtained a marketing authorization valid throughout the European Union and the United states granted by the European Commission and Food and Drug Administration in 2009.

Regarding pharmacokinetic properties, following a single oral or intravenous dose, dronedarone is extensively metabolized in humans to a variety of different metabolites which are excreted in urine and feces. Dronedarone was only observed in low amount in feces from subjects dosed orally, indicating that it is extensively absorbed and metabolized. Major pathways of dronedarone metabolism included *N*-debutylation, followed by oxidation; *N*-dealkylation (or deamination) to form the propanoic acid metabolite, followed by oxidation; and direct oxidation of dronedarone.

The majority of dronedarone is eliminated by first-pass metabolism in the liver by CYP3A4 enzymes (>84%). *N*-debutyl-dronedarone, formed primarily by CYP3A4, is a major circulating metabolite with similar plasma exposure than dronedarone and has been demonstrated to be pharmacologically active although with a lower potency than that of the parent drug.

There is little published clinical information on either the metabolism of dronedarone or reported drug-drug interactions (DDI) (Schweizer et al. 2011, http://www.medicines.org.uk/EMC/medicine/22894/SPC/Multaq+400mg+tablets). In vitro, dronedarone has been demonstrated to be mainly metabolized by the CYP3A4 isoform. These in vitro data have been supported by clinical findings: after single dose, the potent CYP3A4 inhibitor ketoconazole increased dronedarone exposure by 25-fold and the potent CYP3A4 inducer rifampicin (600 mg once daily for 7 days) led to a 5-fold lower dronedarone exposure. Dronedarone was also reported as a moderate reversible inhibitor of CYP3A4 and potent reversible inhibitor of CYP2D6 isoforms in vitro in human liver microsomes, with Ki of 41 and 5 *μ*mol/L, respectively. Thus, in a kidney-transplant patient, trough sirolimus concentrations, a sensitive CYP3A substrate, were increased up to threefold following a 3 day-treatment with twice daily 400 mg dronedarone (Tichy et al. 2010) while in the clinical interaction study performed in human healthy volunteers, dronedarone at the dose of 400 mg BID, increased by 2- to 4-fold steady state concentrations of simvastatin and its active metabolite. Additionally, co-administration of dronedarone (400–800 mg twice a day) with metoprolol (200 mg daily) increases steady state exposure by 1.6- to 2.3-folds of the latter in CYP2D6 extensive metabolizers and induces an additive dronedarone dose-dependent negative inotropic effect (Damy et al. 2004).

Although the main *N*-debutyl metabolite of dronedarone (Xie et al. 2011) has also been demonstrated to be active and to have the potential to inhibit CYP2C9, CYP2C19, and CYP1A2, little has been published on the overall metabolic pathway of dronedarone and its primary metabolites (including *N*-debutyl-dronedarone, propanoic acid dronedarone, and phenol-dronedarone) as well as the major enzymes systems and isoforms involved in these specific pathways, including cytochrome P450s (CYPs) (Shimada et al. 1994; Nelson et al. 1996), flavin monooxygenases (FMOs) (Lawton et al. 1994; Phillips et al. 1995), monoamine oxidase (MAOs) (Dorris 1982), and UDP-glucuronosyl-transferase (UGTs) (Mackenzie et al. 1997).

The objectives of this study were to gain a comprehensive understanding of the routes of dronedarone hepatic metabolism. The metabolites were characterized by high-resolution liquid chromatography/tandem mass spectrometry (LC/MS-MS) and by comparison of their retention times on high-performance liquid chromatography (HPLC) and MS spectra with those of reference standards. In addition, by incubation with human liver microsomes, cDNA-expressed CYPs, UGTs, FMOs, and MAOs or human hepatocytes, the products of dronedarone metabolism and the relative contributions of the enzymes involved were determined in the overall cascade of metabolite formation. CYP3A4 was found to be primarily responsible for the formation of *N*-debutyl-dronedarone, but additional minor pathways were also demonstrated. These data provide a good understanding of dronedarone metabolic pathways and more insight on consequences of DDI involving dronedarone.

## Materials and Methods

### Compounds

Dronedarone, *N*-debutyl-dronedarone, propanoic acid-dronedarone, phenol-dronedarone, and *C*-dealkyl-dronedarone were synthesized by the Isotope Chemistry and Metabolite Synthesis Entity of the Drug Disposition Domain of Sanofi (Chilly-Mazarin, France). Their chemical structures are shown in Figure 1.

Midazolam (MDZ), 1′-hydroxy-midazolam (1′-OH-MDZ), tolbutamide, 4-hydroxy-tolbutamide, dextromethorphan, dextrorphan, phenacetin and *O*-deethyl-phenacetin, dimethyl sulfoxide (DMSO), 1-Aminobenzotriazole (ABT), ketoconazole, clorgyline, alamethicin, ethanolamine, transferrin, linoleic acid, ascorbic acid, insulin, *L*-arginin, and glucagon were obtained from Sigma (St Louis, MO). Ham F12 and Williams E media, *L*-glutamine, 4-(2-hydroxyethyl)-1-piperazineethanesulfonic acid (HEPES), sodium pyruvate, penicillin, and streptomycin were purchased from Gibco (Paisley, UK). The 1′-hydroxy-midazolam glucuronide (Glu-O*-*MDZ) was synthesized by the Isotope Chemistry and Metabolite Synthesis Department of Sanofi (Chilly-Mazarin, France). All other chemicals and solvents used were of analytical grade.

### Recombinant human enzymes

Recombinant human CYPs (Supersomes™; BD Gentes, Woburn, MA: CYP1A1, CYP1A2, CYP2A6, CYP2B6, CYP2C8, CYP2C9, CYP2C18, CYP2C19, CYP2D6, CYP2E1, CYP3A4 and CYP3A5), FMOs (Supersomes™: FMO1, FMO3, FMO5), MAOs (Supersomes™: MAO-A and MAO-B), and UGTs (Supersomes™: UGT1A1, UGT1A3, UGT1A4, UGT1A6, UGT1A7, UGT1A8, UGT1A9, UGT1A10, UGT2B4, UGT2B7, UGT2B15, and UGT2B17) expressed in baculovirus-infected insect cells and control microsomes from insect cells infected with wild-type baculovirus were all obtained from BD Gentest (Woburn, MA).

### Human liver microsomes and human hepatocytes

Human hepatic microsomal fractions were obtained from a large pool of human liver (150 individuals) prepared by BD Gentest-Biosciences (BD UltraPool™ HLM 150 – catalogue reference, 452118 Easy Count Box, Woburn, MA). This pooled human liver microsomal batch was also validated internally by the use of FDA-recommended CYP probes (FDA guidance for Industry, draft guidance February 2012).

Cryopreserved human hepatocytes were obtained from BD Gentest, Invitrotechnologies/Celsis (Baltimore, MD) and Gibco/Lifetechnologies (Paisley, UK). Available demographic information for patients including gender and age is reported in Table S1. Cryopreserved human hepatocytes were internally validated by evaluating their viability, plating efficiency and metabolic capacity. Human hepatocyte characteristics are reported in Table S2.

### Primary culture of human hepatocytes

Cryopreserved human hepatocytes were plated on 48-well collagen-coated plastic plates in a chemically defined medium adapted from Isom and Georgoff (1984), consisting of a 50/50 (v/v) mixture of Ham F12/Williams E medium supplemented with 10% decomplemented fetal calf serum, 10 mg/L insulin, 0.8 mg/L glucagon and antibiotics (100 IU penicillin and 100 *μ*g/mL streptomycin). After 4–6 h at 37°C in a 5% CO_2_ and 100% humidified atmosphere, period during which hepatocytes attached to the collagen-matrix, the plating medium was removed and replaced by the same serum-free culture medium supplemented with HEPES (3.6 g/L), ethanolamine (4 mg/L), transferrin (10 mg/L), linoleic acid albumin (1.4 mg/L), glucose (252 mg/L), sodium pyruvate (44 mg/L), ascorbic acid (50 mg/L), arginine (104 mg/L), and L-glutamine (0.7 g/L).

### Drug metabolism assay in human liver microsomes and recombinant CYP, FMO, MAO, and UGT enzymes

For oxidative pathways, the assay mixture was composed of human liver microsomes (1 mg·m·L^−1^) or microsomes of baculovirus-infected cells expressing human enzymes (100 pmol·mL^−1^ for CYPs and 1 mg·mL^−1^ for FMOs and MAOs), nicotinamide adenine dinucleotide phosphate reduced form (NADPH) (1 mmol/L), 100 mmol/L potassium phosphate (pH 7.4), and 5 *μ*mol/L of the investigated compound dissolved in DMSO in a final volume of 500 *μ*L.

The final concentration of DMSO in the reaction mixture was less than 1% (v/v). Initial rate conditions with respect to time and protein concentration for both the disappearance of unchanged drug and the formation of respective metabolites were established in preliminary studies. The enzyme reaction was initiated by addition of NADPH (10 mmol/L aqueous solution) to achieve a final concentration of 1 mmol/L for oxidative CYP- and FMO-dependent reactions or by the drug for MAO-dependent reactions, incubated at 37°C and stopped after 0–30 min by addition of one volume of ice-cold acetonitrile.

For glucuronidation pathways, the assay mixture was composed of human liver microsomes (2 mg·mL^−1^) or microsomes of baculovirus-infected cells expressing human UGTs (1 mg·mL^−1^), uridine 5′-diphosphoglucuronic acid (UDPGA) (3 mmol/L), 100 mmol/L Tris HCl (pH 7.4) containing 5 mmol/L MgCl_2_ and 5 *μ*mol/L of the investigated compound dissolved in DMSO in a final volume of 500 *μ*L. The final concentration of DMSO in the reaction mixture was less than 1% (v/v). Initial rate conditions with respect to time and protein concentration for both the disappearance of unchanged drug and the formation of respective metabolites were established in preliminary studies. The microsomal mixture was first incubated for 10 min at 4°C in the presence of alamethicin (0.125 mg·mL^−1^), then the reaction was initiated by addition of UDPGA (30 mmol/L aqueous solution) to achieve a final concentration of 3 mmol/L for UGT-dependent reactions, incubated at 37°C and stopped after 0–30 min by addition of one volume of ice-cold acetonitrile.

After removal of the proteins by centrifugation at 10,000 rpm for 10 min, a portion of the supernatant was analyzed by LC-UV-MS.

### Metabolic studies using cryopreserved human hepatocytes in primary culture

Experiments were performed in 48-well plastic plates coated with rat tail Collagen type I. Plates were seeded with 0.16 × 10^5^ hepatocytes per well in a final volume of 0.2 mL and incubated at 37°C under a 95% humidified air and 5% CO2 atmosphere. After 3 h during which cells were allowed to attach, the medium was renewed with 0.1 mL serum-free medium. Then, the investigated drug or metabolic probe(s) were added directly to the incubation medium in the absence or the presence of inhibitors such as either ketoconazole as a specific and potent CYP3A inhibitor (3 *μ*mol/L final concentration), clorgyline (0.25 *μ*mol/L final concentration) as a potent MAO-A inhibitor or ABT as a potent but nonselective CYP isoforms inhibitor (1 mmol/L final concentration) (Fabre et al. 1988; Bourrié et al. 1996; Klieber et al. 2008). Regardless of the final concentration investigated, the final solvent (DMSO) concentration never exceeded 0.2% (v/v).

To determine the metabolism of the different compounds (metabolic probes, dronedarone, *N*-debutyl-dronedarone, propanoic acid-dronedarone, phenol-dronedarone, and *C*-dealkyl-dronedarone), kinetic studies were performed over 0–24 h. For each time point, 0.1 mL of acetonitrile was added to the specific well for protein precipitation, and both extracellular medium and the cell fraction were scraped together. Cell extracts were transferred to a glass tube and stored at −20°C until analysis. Before analysis, cell homogenates were sonicated for a few seconds, homogenized and centrifuged at 6000 g for 10 min. Supernatant fluids were then analyzed for the different drugs and their specific metabolites.

### LC/MS-MS analysis for drugs and metabolite quantification

LC/MS-MS analysis was performed using an Acquity UPLC System I-Class equipped with a Waters Acquity UPLC BEH C_18_ column (2.1 mm i.d. × 100 mm length, 1.7 *μ*m particle size) coupled to a Xevo TQS mass spectrometer (all from Waters, Milford, MA) used in electrospray ion positive mode.

### LC/MS-MS analysis for metabolite identification

Dronedarone and its metabolites were analyzed using Thermo Fisher Scientific Instruments (Waltham, MA): Accela LC system consisted of a degasser, a quaternary pump and an HTC PAL autosampler (CTC Analytics) coupled to a Finnigan LTQ-Orbitrap instrument with an electrospray ionization mode (ESI) source and HCD collision cell. The separation was performed on an YMC-pack J’sphere H80 (250 mm × 2.1 mm, i.d., 4 *μ*m) column and the column temperature was set at 45°C. Gradient elution was performed with 5 mmol/L aqueous ammonium acetate buffer containing 0.1% (v/v) formic acid as mobile phase A and acetonitrile as mobile phase B. The flow rate was 350 *μ*L·min^−1^. Separation of dronedarone and its metabolites was achieved using programmed linear changes in mobile phase composition with increasing phase B from 5 to 70% between 0 and 50 min, followed by a second linear gradient from 70% to 95% of phase B in 0.1 min, which was maintained for 5 min to ensure complete elution of nonpolar metabolites. The injection volume was 50 *μ*L.

The MS conditions were as follows: positive scan mode from *m/z* 100 to 1500 (full scan mass spectrum at a resolution of 30,000); sheath gas, nitrogen at a flow rate of 30 arbitrary units (AU); auxiliary gas, nitrogen at flow rate of 15 AU; spray voltage, 5.00 kV; ion transfer capillary temperature, 250°C; maximum injection time, 500 msec; HCD at 35 eV (MS/MS, positive scan mode from *m/z* 100 to 1500, resolution 30,000).

The instrument was mass calibrated prior to analysis infusing a Positive Mode Cal Mix provided by Supelco at a flow rate of 5 *μ*L·min^−1^ using a syringe pump.

For all detected metabolites, the calculated and measured exact masses and their delta values in ppm as well as their predominant fragments using HR-ESI-MS (MS scan without HCD fragmentation, MS^2^ scan after HCD fragmentation 35 eV) are reported in Table 3.

**Figure 2:**
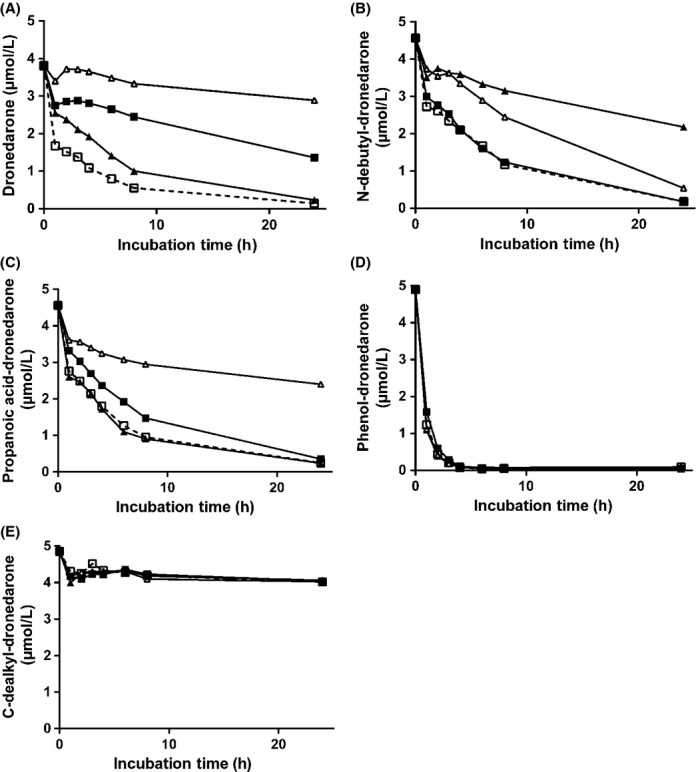
Kinetics of unchanged drug disappearance following incubation of primary cultures of human hepatocytes with either dronedarone (A) or its metabolites, that is *N*-debutyl-dronedarone (B); propanoic acid-dronedarone (C); phenol-dronedarone (D) or *C*-dealkyl-dronedarone (E), at a concentration of 5 *μ*mol/L, in the absence (control, □-□) or presence of different inhibitors (ketoconazole, ▪-▪; 1-aminobenzotriazole, Δ-Δ; clorgyline, ▲-▲). Incubated drugs were quantified by LC/MS-MS relative to the synthetic references as described in Materials and Methods. Results are expressed as the mean unchanged drug concentration of three or four different human hepatocyte preparations.

These analytical conditions allowed an efficient separation of dronedarone from its different metabolites (Fig. 2).

### Data analysis

In human liver microsomes and recombinant isoforms, the percentage of metabolism was determined on the basis of unchanged drug disappearance, using the following equation

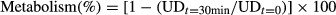
where UD_*t* = 30 min_ represents the unchanged drug peak area after a 30-min incubation period and UD_*t* = 0_, the unchanged drug peak area at *t* = 0.

In human hepatocytes, the intrinsic in vitro metabolic clearances (*Cl*_*int*_) were calculated using WinNonLin PK analysis software version 5.2. All disappearance kinetics data were fitted with a model using the following equation:

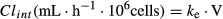
where *k*_e_ is the elimination rate constant expressed in h^−1^, and V is the incubation volume expressed in mL normalized to 10^6^ hepatocytes. The equation was applied to the initial (linear) part of the concentration–time curve.

The fraction metabolized (*f*_m_) by MAO, CYP3A or total CYPs was determined using the following equation:

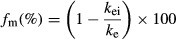
where: *k*_ei_ is the elimination rate constant (expressed in h^−1^) in the presence of the inhibitor.

## Results

### In vitro quantitative biotransformation of dronedarone and its metabolites by cryopreserved human hepatocytes in primary culture

The kinetics of drug disappearance were investigated using four different cryopreserved human hepatocyte preparations in primary cultures following incubation with either dronedarone or its metabolites at a concentration of 5 *μ*mol/L. Incubations were performed either in the absence, that is control conditions, or in the presence of enzyme system inhibitors such as 3 *μ*mol/L ketoconazole (specific and potent inhibitor of CYP3A4/5), 1 mmol/L ABT (a potent and specific inhibitor of total CYPs), or 0.25 *μ*mol/L clorgyline (a potent MAO-A inhibitor) (Dorris 1982). Kinetic studies were performed over 24 h.

Figure 2A–E illustrates the mean (*n* = 3 to 4) kinetics of unchanged drugs disappearance in the absence or the presence of the different inhibitors. Metabolic clearances are reported in Table 1. Dronedarone was metabolized at a high rate (Fig. 2A) with more than 80% of the drug being metabolized within 6 h of incubation. Limited inter-human hepatocyte preparation variability was observed with an in vitro intrinsic metabolic clearance of 0.33 ± 0.21 mL·h^−1^·10^6^ cells. The different metabolites of dronedarone were cleared at different rates, with phenol-dronedarone (Fig. 2D, *Cl*_int_ = 0.61 ± 0.12 mL·h^−1^·10^6^ cells) being extensively metabolized in comparison to *N*-debutyl- and propanoic acid-dronedarone derivatives (Fig. 2B and C, respectively) which exhibited *Cl*_int_ at 0.13 ± 0.03 and 0.14 ± 0.05 mL·h^−1^·10^6^ cells, respectively. There was very little metabolism of *C*-dealkyl-dronedarone under these experimental conditions (Fig. 2E) with a very low clearance of 0.003 ± 0.001 mL·h^−1^·10^6^ cells.

**Table 1:** Individual metabolic clearance values for dronedarone and its metabolites in each of the different cryopreserved human hepatocyte preparations

	In vitro intrinsic hepatic clearance (mL·h^−1^·10^6^ cells)				
	
Drug	BD-91	BD-296	HU-1359	IVT-IBG	Mean ± SD
Dronedarone	0.49	0.58	0.14	0.10	0.33 ± 0.21
*N*-debutyl-dronedarone	0.18	0.10	0.10	0.08	0.13 ± 0.03
Propanoic acid-dronedarone	0.06	0.17	0.20	0.14	0.14 ± 0.05
Phenol-dronedarone	0.44	0.70	0.57	0.75	0.62 ± 0.12
*C*-dealkyl-dronedarone	0.003	0.002	0.004	0.003	0.003 ± 0.001

Metabolism of dronedarone and its metabolites investigated in the presence of different inhibitors provided an evaluation of the overall contribution of all the CYPs (in the presence of ABT), of CYP3A4/5 (in the presence of ketoconazole) and of MAO-A (in the presence of clorgyline). As illustrated in Figure 2A–E, their inhibitory effects differed markedly as a function of the investigated drug. The contributions of these different enzyme systems or isoforms to the overall in vitro metabolic clearance of dronedarone and its metabolites are reported in Table 2.

**Table 2:** Contribution (fm expressed in percentage) of CYPs (based on 1-aminobenzotriazole inhibitory effect), CYP3A4 (based on ketoconazole inhibitory effect) and MAO (based on clorgyline inhibitory effect) to the individual metabolic clearance of dronedarone and its metabolites in each of the four different cryopreserved human hepatocyte preparations

*f*_m_	BD-91	BD-296	HU-1358	IVT-IBG	Mean ± SD
Dronedarone					
Total CYPs (%)	98	99	98	95	98 ± 2
CYP3A4 (%)	95	95	84	80	89 ± 7
MAO (%)	18	–	23	43	29 ± 14
*N*-debutyl-dronedarone					
Total CYPs (%)	67	48	51	54	55 ± 7
CYP3A4 (%)	7	4	8	0	5 ± 3
MAO (%)	88	80	86	83	84 ± 3
Propanoic acid-dronedarone					
Total CYPs (%)	87	92	91	89	90 ± 3
CYP3A4 (%)	36	41	33	34	36 ± 3
MAO (%)	12	0	0	0	3 ± 5

#### Dronedarone

ABT and ketoconazole exhibited a predominant inhibitory effect demonstrating the major contribution of the CYPs and more precisely CYP3A4/5 isoforms in dronedarone clearance (Fig. 2A). Clorgyline exhibited a minor inhibitory effect.

**Figure 3:**
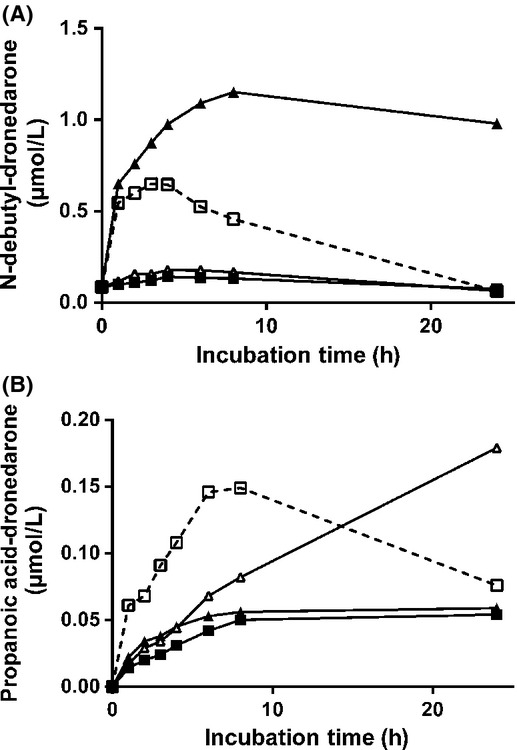
Kinetics of *N*-debutyl-dronedarone (A) and propanoic acid-dronedarone (B) appearance following incubation of primary cultures of human hepatocytes with 5 *μ*mol/L dronedarone alone (control, □-□) or in the presence of different inhibitors (ketoconazole, ▪-▪; 1-aminobenzotriazole, Δ-Δ; clorgyline, ▲-▲). Metabolites were quantified by LC/MS-MS relative to the synthetic references as described in Materials and Methods. Results are expressed as the mean concentrations of four different human hepatocyte preparations.

In addition to drug disappearance, the quantitative formation of reference metabolites was investigated. The kinetics of *N*-debutyl-dronedarone and propanoic acid-dronedarone formation following incubation of primary cultures of human hepatocytes with dronedarone in the absence or the presence of the various CYPs and MAO-A inhibitors are presented in Figure 3A and B, respectively. Under control conditions without inhibitors, *N*-debutyl-dronedarone concentration rapidly increased to achieve a mean maximal concentration of around 0.6 *μ*mol/L, and then its concentration continually decreased, suggesting its own biotransformation. *N*-debutyl-dronedarone formation was almost fully inhibited in the presence of both ketoconazole and ABT demonstrating the predominant contribution of CYP3A4/5 in its formation. No effect was observed on the initial rate of *N*-debutyl-dronedarone formation in the presence of clorgyline, the MAO-A inhibitor. The concentration of *N*-debutyl-dronedarone continually increased up to the 24th hour suggesting a role for MAO-A in its further metabolism. Propanoic acid-dronedarone formation was also investigated following incubation of human hepatocytes with dronedarone (Fig. 3B). Under control conditions, its concentration increased to achieve a maximal concentration between 6 and 8 h, and then slowly decreased over the remaining period of incubation. Its rate of formation was potently inhibited by CYP and MAO-A inhibitors, while its rate of further metabolism was only inhibited by ABT suggesting the contribution of CYPs other than CYP3A4/5 in its further metabolism.

#### *N*-debutyl-dronedarone

**Figure 4:**
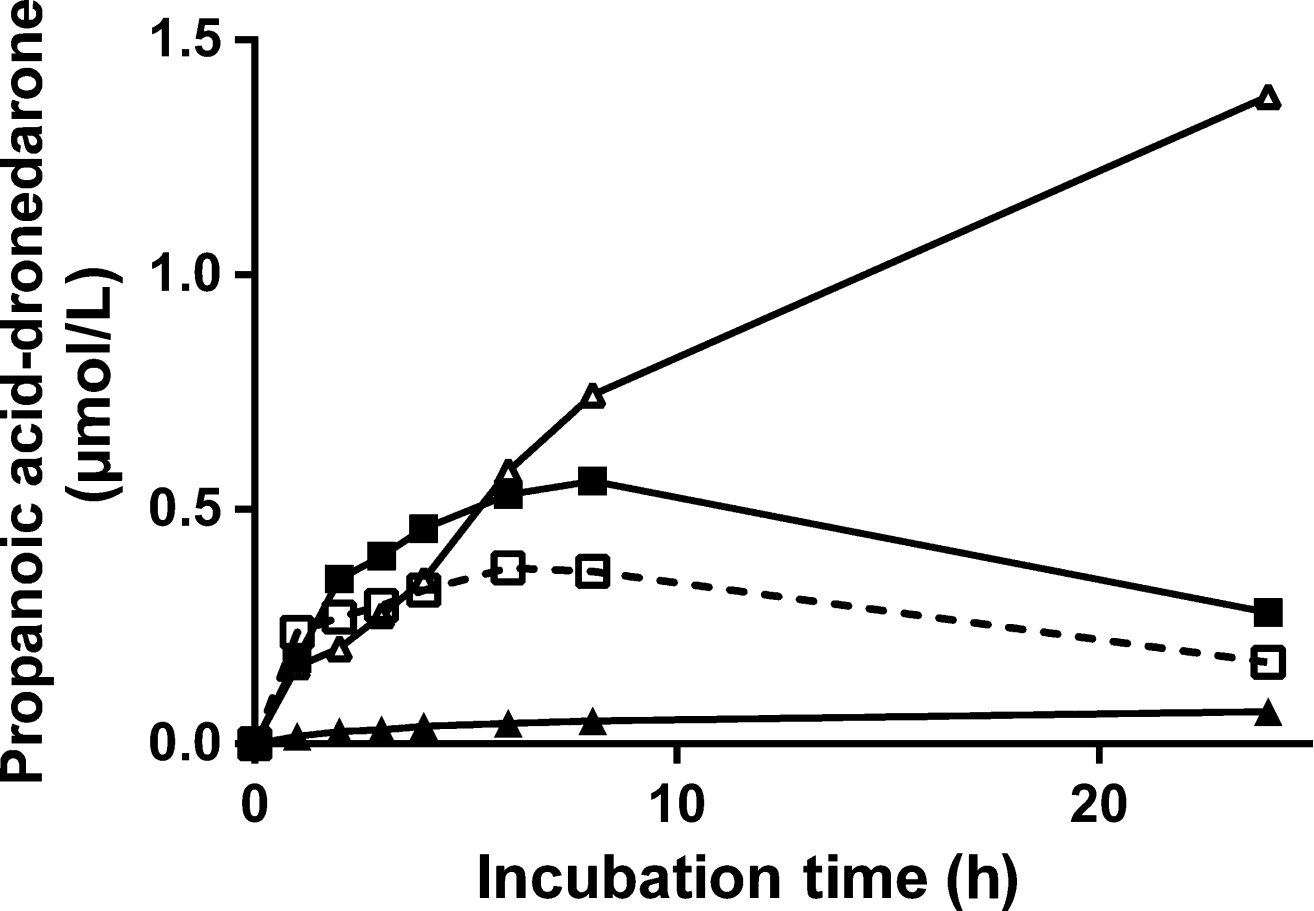
Kinetics of propanoic acid-dronedarone appearance following incubation of primary cultures of human hepatocytes with 5 *μ*mol/L *N*-debutyl-dronedarone alone (control, □-□) or in the presence of different inhibitors (ketoconazole, ▪-▪; 1-aminobenzotriazole, Δ-Δ; clorgyline, ▲-▲). Propanoic acid-dronedarone was quantified by LC/MS-MS relative to the synthetic reference as described in Materials and Methods. Results are expressed as the mean concentrations of four different human hepatocyte preparations.

Ketoconazole exhibited no inhibitory effect while ABT moderately decreased its metabolic rate suggesting the involvement of CYPs other than CYP3A4/5 as well as other enzyme systems to *N*-debutyl-dronedarone in vitro hepatic clearance (Fig. 2B). *N*-debutyl-dronedarone metabolism was potently inhibited by the MAO-A inhibitor, clorgyline. Additional data also demonstrated that deprenyl (a non-specific MAO inhibitor) was as potent as clorgyline in inhibiting *N*-debutyl-dronedarone metabolism (data not shown). Taken together, these data suggested that MAO-A was predominantly involved in *N*-debutyl-dronedarone metabolic clearance. Propanoic acid-dronedarone was also quantified following incubation of human hepatocytes with *N*-debutyl-dronedarone with or without CYP and MAO-A inhibitors (Fig. 4). The rate of propanoic acid-dronedarone formation was only slightly modified by CYP inhibitors (both ketoconazole and ABT), but potently inhibited by clorgyline demonstrating the predominant role of MAO-A in its formation. Although ABT did not contribute to propanoic acid-dronedarone formation, it clearly inhibited its metabolism, as demonstrated by a continuous accumulation of propanoic acid-dronedarone over the 24-h incubation period.

#### Propanoic acid-dronedarone

Clorgyline had no inhibitory effect upon the metabolism of propanoic acid dronedarone, while ketoconazole only exhibited a very minor inhibitory effect. In contrast propanoic acid-dronedarone metabolism was potently inhibited by ABT, demonstrating the major contribution of CYP isoforms, other than CYP3A4/5, to its metabolism (Fig. 2C).

#### Phenol-dronedarone

Incubation with relevant CYP and MAO inhibitors indicated that these enzyme systems did not contribute to its in vitro metabolism (Fig. 2D).

#### *C*-dealkyl-dronedarone

It exhibited a very low hepatic clearance, and no effect of any of the inhibitors could be detected (Fig. 2E).

### In vitro qualitative biotransformation of dronedarone and its metabolites by cryopreserved human hepatocytes in primary culture

Pooled human hepatocyte samples (*n* = 4) obtained following a 6-h incubation with dronedarone and its metabolites were analyzed by LC/MS-MS in order to identify the different metabolites. Mass spectra and analytical characteristics as well as proposed structures for all major metabolites identified following incubation of dronedarone and its metabolites with different in vitro models are reported in Table 3.

**Table 3:** Assignment, proposed structures, and MS/MS characteristics of major metabolites identified following incubation of either human liver microsomal fractions (1 mg/mL final concentration; in the absence or presence of either 1 mmol/L NADPH or 3 mmol/L UDPGA), recombinant human isoforms (CYPs, FMOs, MAO, and UGTs) or human hepatocytes (pool of four preparations in the absence or presence of CYP and MAO inhibitors) with dronedarone and its metabolites (5 *μ*mol/L final concentration)

Assign^ment^	Rt (min)	Metabolite description	Theoretical MH^+^ *(m/z)*	Measured MH^+^ *(m/z)*	ΔM ppm	Elemental composition MH^+^ *(m/z)*	Product ions *(m/z)*
M1	19.5	Hydroxy phenol-dronedarone glucuronide	580.14832	580.14879	0.806	C_26_H_30_NO_12_S^+^	121, 386, 404
M2	20.0	*C*-dealkyl-dronedarone	308.22202	308.22217	0.486	C_18_H_30_NO_3_^+^	114, 170, 252
M3	20.7	Hydroxy phenol-dronedarone glucuronide	580.14832	580.14836	0.065	C_26_H_30_NO_12_S^+^	121, 386, 404
M4	22.6	Hydroxy *N*-debutyl-dronedarone	517.23668	517.23650	−0.356	C_27_H_37_NO_6_S^+^	86, 114, 386
M5	23.3	Oxidized *N*-debutyl-dronedarone	515.22103	515.22180	1.487	C_27_H_35_N_2_O_6_S^+^	86, 114, 308
M6	23.4	Dihydroxy-dronedarone	589.29420	589.29528	1.835	C_31_H_45_N_2_O_7_S^+^	158, 186, 467
M7	23.8	Hydroxy *N*-debutyl-dronedarone	517.23668	517.23638	−0.588	C_27_H_37_N_2_O_6_S^+^	86, 114, 294, 386
M8	25.2	Hydroxy phenol-dronedarone	404.11623	404.11663	0.979	C_20_H_22_NO_6_S^+^	121
M9	25.7	Hydroxy, oxidized propanoic acid-dronedarone	490.11663	490.11684	0.432	C_23_H_24_NO_9_S^+^	193
M10	25.8	Hydroxy propanoic acid-dronedarone	476.13736	476.13742	0.118	C_23_H_26_NO_8_S^+^	193
M11	26.7	Hydroxy propanoic acid-dronedarone	476.13736	476.13727	−0.197	C_23_H_26_NO_8_S^+^	193
M12	26.9	Hydroxy dronedarone	573.29928	573.29883	−0.792	C_31_H_45_N_2_O_6_S^+^	114, 142, 170, 451, 517
M13	27.1	*N*,*N*′-Didebutyl dronedarone	445.17917	445.17947	0.676	C_23_H_29_N_2_O_5_S^+^	58, 294, 388
M14	27.4	Hydroxy dronedarone	573.29928	573.29895	−0.583	C_31_H_45_N_2_O_6_S^+^	114, 142, 170, 386, 451, 517
M15	27.6	*O*-dealkylation hydroxyl dronedarone	404.11623	404.11586	−0.927	C_20_H_22_NO_6_S^+^	121, 292
M16	28.3	Hydroxy *N*-debutyl-dronedarone	517.23668	517.23676	0.108	C_27_H_37_N_2_O_6_S^+^	102, 130, 294, 388
M17	29.1	Dihydroxy-dronedarone	589.29420	589.29326	1.593	C_31_H_45_N_2_O_7_S^+^	174, 202, 294, 451
M18	29.2	Hydroxy phenol-dronedarone	404.11623	404.11639	0.385	C_20_H_22_NO_6_S^+^	121, 292
M19	29.9	Phenol-dronedarone glucuronide	564.15341	564.15289	−0.918	C_26_H_30_NO_11_S^+^	121, 294, 388
M20	30.2	Hydroxy propanoic acid-dronedarone	476.13736	476.13782	0.958	C_23_H_26_NO_8_S^+^	193, 292, 458
M21	31.4	*N*-debutyl-dronedarone	501.24177	501.24172	−0.099	C_27_H_37_N_2_O_5_S^+^	86, 114, 294, 388
M22	32.4	Hydroxy dronedarone	573.29928	573.29924	−0.077	C_31_H_45_N_2_O_6_S^+^	158, 186, 294, 451
M23	32.8	Deaminated *N*,*N*′-didebutyl dronedarone glucuronide	622.19527	622.19434	−1.499	C_29_H_36_NO_12_S^+^	294, 446
PD	36.3	Dronedarone	557.30437	557.30437	0.001	C_31_H_45_N_2_O_5_S^+^	114, 142, 170, 294, 435, 501
M24	38.5	Phenol-dronedarone	388.12132	388.12152	0.515	C_20_H_22_NO_5_S^+^	121, 294
M25	39.1	Propanoic acid-dronedarone	460.14245	460.14296	1.110	C_23_H_26_NO_7_S^+^	193, 294
M26	39.5	Deaminated *N*,*N*′-didebutyl dronedarone	446.16318	446.16387	1.536	C_23_H_28_NO_6_S^+^	121, 179, 294

HRMS, high-resolution mass spectrometry; Assign^ment^, assignment; Rt, retention time; PD, parent drug.

#### Dronedarone

The disappearance of dronedarone (as illustrated in Fig. S1A) was associated with the formation of numerous metabolites obtained by *N*-debutylation (mono and di-debutylated i.e. M21 and M13, respectively), hydroxylation on the dibutylamine moiety (M22), *O*-dealkylation (M24), deamination (M25) and different combinations of these different processes such as hydroxylation and *N*-debutylation (M4 and M16) and dihydroxylation (M6 and M17). When incubated in the presence of enzyme inhibitors, major changes in the metabolic profiles were observed. In the presence of clorgyline (Fig. S1D), only a minor increase in unchanged dronedarone was observed, most noticeable differences being the almost complete disappearance of the propanoic acid (M25) metabolite and an increase in *N*-debutyl-dronedarone (M21). Much larger differences were observed following co-incubation with CYP inhibitors. In the presence of ketoconazole (Fig. S1B), a significant increase in unchanged dronedarone was observed, associated with a decrease in the *N*-debutyl-hydroxyl metabolite (M16) and a large increase in M14 (hydroxyl on the butyl-benzofuran moiety). Following co-incubation with ABT (Fig. S1C), most of the metabolite levels decreased markedly or disappeared, except M22 (hydroxylation on the dibutylamine) whose concentration increased. All these data suggest that *N*-debutylation (M21) clearly appears to be CYP3A4/5-dependent, and hydroxylation on the butyl-benzofuran moiety (M14) is CYP-dependent but not associated with CYP3A4/5, while hydroxylation on the dibutylamine moiety (M22) does not appear to be CYP-dependent.

#### *N*-debutyl-dronedarone

Ion current chromatograms obtained under control conditions are shown in Figure S2A. Major metabolites were obtained by either direct oxidation (M5) or hydroxylation (M4) on the butyl-benzofuran moiety, *O*-dealkylation (M24) or deamination (M25). The latter metabolite was metabolized further to the hydroxyl (M11) and subsequently to the carboxylic acid (M9 derivative). When *N*-debutyl-dronedarone was incubated in the presence of ketoconazole, only minor changes were observed (Fig. S2B), which was in contrast to the effect of ABT (Fig. S2C). Under the latter CYP-inhibition conditions, all metabolites disappeared, except the propanoic acid derivative (M25), the phenol derivative (M24) and its glucuronic acid conjugate (M19), demonstrating that the formation of these metabolites was not CYP-dependent. This was further confirmed by the metabolite profiles generated in the presence of clorgyline (Fig. S2D), in which the complete disappearance of the, propanoic acid derivative (M25), phenol derivative (M24) and their hydroxylated (M11), oxidized (M9) and glucuronidated (M19) derivatives were observed. This was associated with an increase in, *N*-debutylation (M13) and direct hydroxylation, either on the butyl-benzofuran (M4) or on the butylamine (M16) moiety.

#### Propanoic acid-dronedarone

The main metabolites detected under control conditions consisted of hydroxylation (M11) and further oxidation (M9) of the butyl-benzofuran moiety (Fig. S3A). Additional minor metabolites were observed (M1 and M3) which correspond to glucuronic acid conjugates of the hydroxylated-phenol derivatives. Following incubation in the presence of ketoconazole (Fig. S3B), only a change in the relative proportion of the two major metabolites was observed, associated with a slight increase in unchanged drug, suggesting that the formation of these metabolites may not be associated with CYP3A4/5. Since the formation of these metabolites was fully inhibited by ABT (Fig. S3C) and not affected by clorgyline (Fig. S3D), CYPs other than CYP3A4/5 are involved in their formation.

#### Phenol-dronedarone

Phenol-dronedarone was extensively metabolized under control conditions into three metabolites, M19 obtained by direct glucuronidation and, M1 and M3, both metabolites being hydroxylated at two different positions of the butyl-benzofuran moiety, and conjugated with glucuronic acid (Fig. S4A). Both ketoconazole and clorgyline had no effect (Fig. S4B and D), while a complete disappearance of M1 and M3 was observed in the presence of ABT (Fig. S4C). Altogether these data suggest that non-CYP3A4/5 isoforms are involved in the primary formation of the two hydroxyl metabolites.

#### *C*-dealkyl-dronedarone

Only trace amounts of *C*-dealkyl-dronedarone (M2) or its metabolites were observed, consistent with the very low metabolic clearance in the different human hepatocyte preparations either in the presence or absence of inhibitors (data not shown). The main metabolic pathways were 1-O-acyl glucuronidation, hydroxylation of the dibutylamine chain and glycine conjugation.

### In vitro oxidative metabolism of dronedarone and its metabolites by human hepatic microsomes

Dronedarone and its metabolites were incubated over 30 min with human hepatic microsomal fractions (1 mg·mL^−1^) at a final concentration of 5 *μ*mol/L, in the absence or the presence of 1 mmol/L NADPH, the cofactor for CYP- and FMO-dependent reactions.

#### Dronedarone

In the absence of NADPH, dronedarone was not metabolized. In the presence of NADPH, Dronedarone was rapidly metabolized, almost 60% of parent drug being metabolized at the 30^th^ min of incubation. LC/MS-MS analysis showed that dronedarone was converted primarily to its *N*-debutyl metabolite (M21). Its chemical structure was confirmed by LC/MS-MS and its retention time matched with the reference standard. In addition to M21, several minor metabolites were also observed and identified as the hydroxyl derivatives (M4 and M7) and phenol-dronedarone (M24). When incubated in the presence of either ketoconazole or ABT, *N*-debutyl metabolite formation was fully suppressed, suggesting that CYP3A4/5 contributed predominantly to dronedarone oxidation (data not shown).

#### *N*-debutyl-dronedarone

*N*-debutyl-dronedarone was metabolized (about 25–30%) both in the absence and presence of NADPH suggesting the contribution of non-CYP-dependent enzymes. Regardless of whether NADPH cofactor was present or not (data not shown), *N*-debutyl-dronedarone was converted into two main metabolites whose retention times and mass characteristics identified them as phenol- (M24; retention time = 38.5 min) and propanoic acid- (M25; retention time = 39.1 min) dronedarone metabolites, respectively. Since these two metabolites were also formed in the absence of NADPH, this clearly demonstrated that the CYP family of enzymes was not involved in their formation. When incubated in the presence of NADPH, additional minor metabolites were observed and identified as the *N*,*N*′-didebutyl (M13) and hydroxyl (M4) derivatives confirming the role of CYP isoforms in their formation.

#### Propanoic acid-dronedarone

No metabolism occurred in the absence of the NADPH cofactor. In the presence of NADPH, propanoic acid-dronedarone was oxidized, 50% of initial drug being metabolized after 30 min. The disappearance of unchanged drug was associated with the formation of various metabolites (M10 and M11) identified as hydroxyl derivatives. Only traces of phenol-dronedarone (M24) were observed on the MS-chromatogram.

#### Phenol-dronedarone

Whatever the absence or presence of NADPH, phenol-dronedarone metabolism was very slow and the disappearance of unchanged drug was mainly associated with the formation of a hydroxyl derivative (M8).

#### *C*-dealkyl-dronedarone

No metabolism was observed in either the absence or presence of NADPH (data not shown).

### Oxidation of dronedarone and its metabolites by recombinant human CYP, FMO and MAO enzymes

To identify the specific isoforms that are capable of oxidizing dronedarone and its metabolites, assays were performed using c-DNA-expressed isoforms belonging to CYP- (CYP1A1, -1A2, -2A6, -2B6, -C8, -2C9, -2C18, -2C19, -2D6, -2E1, -3A4, and -3A5), FMO- (FMO-1, -3, and -5) and MAO- (MAO-A and MAO-B) families.

#### Dronedarone

**Figure 5:**
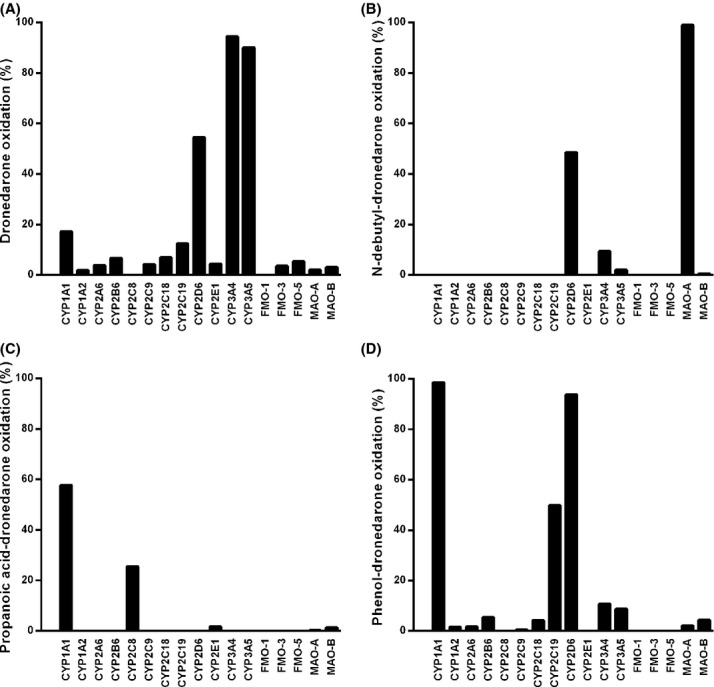
Contribution of individual CYPs, FMOs and MAOs to the oxidation of dronedarone and its metabolites. Individual CYPs (100 pmol/mL), FMOs (1 mg/mL) and MAOs (1 mg/mL) were incubated with either dronedarone (A) or its metabolites, *N*-debutyl-dronedarone (B), propanoic acid-dronedarone (C), and phenol-dronedarone (D), at a concentration of 5 *μ*mol/L, for 30 min at 37°C in the presence of NADPH, except for MAOs where incubations were performed in the absence of cofactor. Unchanged drugs were quantified by LC/MS-MS relative to the synthetic references as described in Materials and Methods. Data are expressed as the percentage of oxidation.

CYP2D6, CYP3A4, and CYP3A5 were the only three CYP isoforms able to oxidize dronedarone. FMOs as well as MAOs were not involved in dronedarone oxidation, as demonstrated by careful LC/MS-MS analysis of all UV-detected peaks (data not shown). Percentages of oxidation, based on the disappearance of unchanged drug, are illustrated in Figure 5A. Both CYP3A4 and CYP3A5 metabolized dronedarone by 94% and 90%, respectively, under these experimental conditions, while only 50% of dronedarone was metabolized by CYP2D6. *N*-debutyl-dronedarone represented 60% with CYP3A4, 50% with CYP3A5 and less than 1% with CYP2D6 of initial dronedarone concentration at the end of the 30-min incubation period, demonstrating that CYP2D6 did not contribute to the formation of *N*-debutyl-dronedarone. Following incubation of dronedarone with CYP2D6, mainly two hydroxyl derivatives (M12 and M14) were observed (data not shown), with the hydroxylation occurring in the butyl-benzofuran moiety. None of the other CYP isoforms were involved in the overall dronedarone oxidation (less than 15% of oxidation after a 30-min incubation period) or in *N*-debutyl-dronedarone formation.

#### *N*-debutyl-dronedarone

Only MAO-A, CYP2D6, and CYP3A4 were able to oxidize *N*-debutyl-dronedarone by 98%, 55% and 21%, respectively (Fig. 5B). Following incubation with CYP2D6, the hydroxyl (M4) and the oxidized derivatives (M5) were the main metabolites while only the *N*,*N*′-didebutyl derivative (M13) was identified with CYP3A4. Neither other CYPs nor FMOs metabolized *N*-debutyl-dronedarone to any significant extent. When incubated with MAO-A, *N*-debutyl-dronedarone completely disappeared and was associated with the simultaneous increase in the formation of the propanoic acid derivative (M24). Of interest is the observation that no metabolism occurred when MAO-B was incubated, suggesting a very high affinity of *N*-debutyl-dronedarone for MAO-A. Control experiments performed with kynuramine, a non-specific-MAO substrate (Dorris 1982), demonstrated the metabolic capacity of both MAO-A and MAO-B (data not shown).

#### Propanoic acid-dronedarone

Only CYP2C8 and CYP1A1 were able to oxidize propanoic acid-dronedarone by 28% and 59%, respectively, over the incubation period (Fig. 5C). Neither other CYPs, nor FMOs and MAOs metabolized propanoic acid-dronedarone to any significant extent. CYP2C8 predominantly hydroxylated propanoic acid dronedarone at different positions on the butyl-benzofuran moiety (M10 and M11).

Although a high oxidation rate was observed with CYP1A1, leading to the formation of two hydroxyl derivatives (M11 and M20), it is likely that this CYP isoform plays a minor role in propanoic acid-dronedarone metabolism by the human liver since it has already been demonstrated that constitutive CYP1A1 is present in only very low amounts in the human liver (Daujat et al. 1990). This hypothesis is in agreement with the observation that M19 was not detected following incubation of human hepatic microsomal fractions with propanoic acid-dronedarone in the presence of NADPH. With CYP1A1 being inducible by potential co-administered drugs and different environmental compounds, CYP1A1 contribution to propanoic acid-dronedarone metabolism in vivo should only appear in CYP1A1-induced patients.

#### Phenol-dronedarone

Only CYP1A1, CYP2C19, and CYP2D6 were able to oxidize phenol-dronedarone with 99%, 52%, and 94% metabolism, respectively during incubation (Fig. 5D). Only hydroxyl derivatives of the butyl-benzofuran moiety (M8, M15 and M18) were formed under these experimental conditions. It has to be noted that CYP1A1 formed mainly M18 and CYP2D6 M8 only, while CYP2C19 formed M8, M15 and M18 in equal relative amounts. All other CYPs, as well as FMOs and MAOs contributed to a very small extent.

#### *C*-dealkyl-dronedarone

This compound was not metabolized by any of the different CYPs, FMOs or MAOs.

### In vitro glucuronidation of dronedarone and its metabolites by human hepatic microsomes

Since a *N*-glucuronidation process could not be excluded, and because some of the metabolites bear either, a free hydroxyl group or carboxylic acid function, glucuronidation of dronedarone and its potential metabolites was also investigated in human hepatic microsomal fractions in the presence of UDPGA as cofactor. Only propanoic acid-dronedarone and phenol-dronedarone were metabolized in the presence of UDPGA.

**Figure 6:**
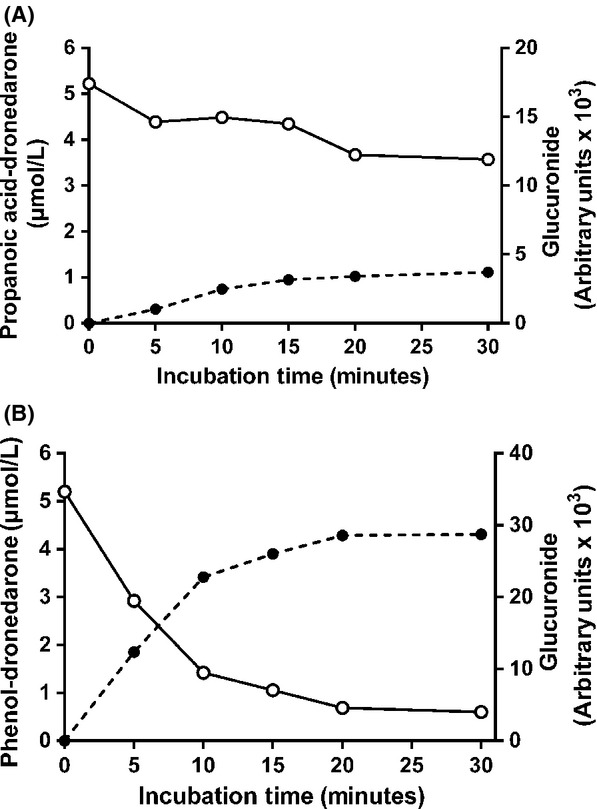
Quantitative glucuronidation of propanoic acid-dronedarone (A) and phenol-dronedarone (B) during a 30-min incubation of human hepatic microsomal fractions (1 mg/mL final concentration) with 5 *μ*mol/L unchanged drug in the presence of UDPGA. The kinetics of propanoic acid-dronedarone disappearance (○-○), phenol-dronedarone disappearance and their respective glucuronide formation (●-●, dashed line) were recorded over 30 min. Propanoic acid-dronedarone and phenol-dronedarone were quantified by LC/MS-MS relative to the synthetic references, while the peak area of their respective identified glucuronide was expressed in arbitrary units (×10^3^), as described in Materials and Methods.

Propanoic acid-dronedarone was poorly metabolized to a major glucuronide, the unchanged drug still representing 70% of initial concentration at the 30th minute of incubation (Fig. 6A). On the other hand, phenol-dronedarone was extensively metabolized with more than 70% of initial drug being metabolized in 10 min (Fig. 6B).

### Quantitative glucuronidation of dronedarone and its metabolites by recombinant human UGT enzymes

Human recombinant UGT isoforms expressed in baculovirus-infected insect cells (UGT1A1, UGT1A3, UGT1A4, UGT1A6, UGT1A7, UGT1A8, UGT1A9, UGT1A10, UGT2B4, UGT2B7, UGT2B15, and UGT2B17) were compared with regard to their ability to catalyze the glucuronidation of dronedarone and its metabolites.

No glucuronidation was observed for dronedarone or its *N*-debutyl and *C*-dealkyl-metabolites.

**Figure 7:**
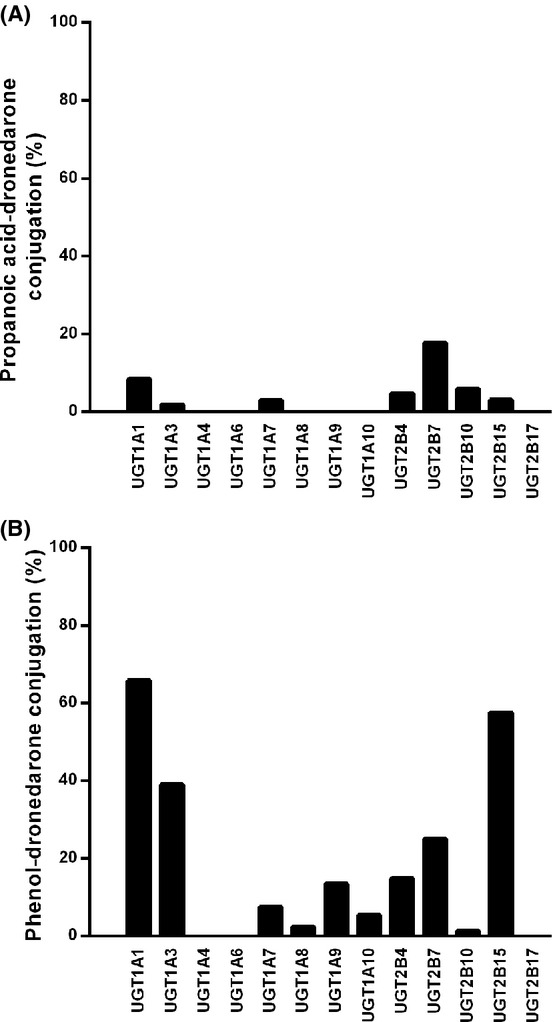
Contribution of individual UGTs to the glucuronidation of propanoic acid-dronedarone and phenol-dronedarone. Individual UGTs (1 mg/mL) were incubated with either propanoic acid-dronedarone (A) or phenol-dronedarone (B), at a concentration of 5 *μ*mol/L, for 30 min at 37°C in the presence of UDPGA. Unchanged drugs were quantified by LC/MS-MS relative to the synthetic references as described in Materials and Methods. Data are expressed as the percentage of glucuronidation.

Among the different isoforms investigated, only recombinant UGT2B7 for propanoic acid-dronedarone (Fig. 7A), and UGT1A1, UGT1A3, UGT2B7, and UGT2B15 for phenol-dronedarone (Fig. 7B) showed any significant glucuronosyl transferase activity.

## Discussion

Dronedarone is a noniodinated benzofuran derivative described as mainly metabolized into its active *N*-debutyl-dronedarone metabolite via a CYP3A-dependent metabolic pathway.

The majority of dronedarone is eliminated by first-pass metabolism in the liver by CYP3A4/5 enzymes, 84% of the dose being excreted in feces mainly as metabolites.

In vitro, dronedarone was extensively metabolized by human liver microsomes and human hepatocytes. These data were in good agreement with the extensive systemic clearance observed in vivo. However, marked differences in metabolic profiles were observed with liver microsomes, recombinant enzymes and hepatocytes, indicating that different enzyme systems as well as isoforms were involved in overall dronedarone metabolism.

Since the major primary metabolites of dronedarone, that is those obtained by *N*-debutylation, *O*-dealkylation, and deamination were chemically synthetized and available, the objective of the study was to elucidate the overall metabolism and metabolic pathways of dronedarone. This was achieved by studying the metabolism of both dronedarone and its major primary metabolites in different in vitro models, such as cryopreserved human hepatocytes incubated in the absence or presence of the specific CYP3A4/5 inhibitor, that is ketoconazole, the total CYP inhibitor, that is ABT, and the MAO-A inhibitor, that is clorgyline, human hepatic microsomal fractions incubated in the absence or presence of different cofactors, NADPH or UDPGA for CYP- together with FMO-dependent or UGT-dependent reactions, respectively, and recombinant human CYP, FMO, MAO, and UGT isoforms.

**Figure 8:**
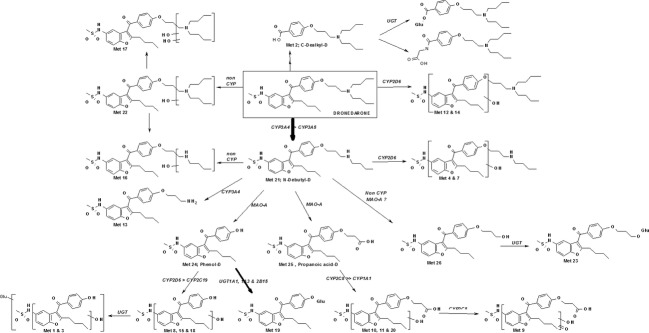
Schematic representation of in vitro metabolism of dronedarone and four of its metabolites: *N*-debutyl-dronedarone, phenol-dronedarone, propanoic acid-dronedarone and *C*-dealkyl-dronedarone. Relevant P450, UGT and MAO enzymes involved in the formation and further metabolism of each of these metabolites are indicated. The size of the arrows is representative of the quantitative metabolic rate based on human hepatocyte clearance data. Detailed incubation procedures using the following biological matrices, hepatocytes, hepatic microsomes and recombinant enzymes are described under Materials and Methods.

This approach has provided a clear picture of the overall human metabolic pathways for dronedarone (Fig. 8), including both the determination of metabolic rates and identification of the enzyme systems involved. The primary metabolites of dronedarone were *N*-debutyl-dronedarone (M21), benzofuran-hydroxyl-dronedarone (M12 and M14), dibutylamine-hydroxyl-dronedarone (M22), and the main Phase II metabolites were derived from phenol-dronedarone, either by direct glucuronidation (M19) or after intermediary hydroxylation (M1 and M3). In addition, both phenol-dronedarone (M24) and propanoic acid-dronedarone (M25) were major metabolites in the overall metabolic cascade of dronedarone.

Following incubation with cryopreserved human hepatocytes, dronedarone was rapidly metabolized in its *N*-debutyl-dronedarone metabolite, a reaction which was almost fully inhibited (89 ± 7%) by ketoconazole, a CYP3A4/5 inhibitor and totally inhibited (98 ± 2%) by ABT, a potent total-CYP inhibitor. These data demonstrated the crucial role of CYP3A4/5 in dronedarone metabolism, and were fully supported by the preliminary screen using 12 recombinant human enzymes (CYP, 3 FMO, and 2 MAO Supersomes). Although dronedarone was mostly *N*-debutylated by CYP3A4/5 isoforms, *N*-debutyl-dronedarone concentrations decreased rapidly (data not shown), suggesting that *N*-debutyl-dronedarone was itself rapidly metabolized. This was confirmed following its incubation with several human hepatocyte preparations demonstrating a major MAO contribution as evidenced by the strong inhibitory effect of clorgyline (84 ± 3%).

In human hepatic microsomes, phenol-dronedarone and propanoic acid-dronedarone were formed in the absence of NADPH cofactor. In the presence of NADPH, hydroxylation of the butyl-benzofuran moiety (M4) and further debutylation (M13) were observed. These data were supported by incubation of *N*-debutyl-dronedarone with both CYP and MAO isoforms: phenol-dronedarone formation being catalyzed by MAO-A, while one hydroxyl and one oxidized derivative (M4 and M5, respectively) were only formed by CYP2D6. Following incubation with CYP3A4, the *N*,*N*′-didebutyl derivative (M13) was the main observed metabolite.

The behavior of phenol-dronedarone and propanoic acid-dronedarone was further investigated in the different in vitro models. Although phenol-dronedarone could be slightly oxidized to hydroxyl derivatives all located on the butyl-benzofuran moiety, ABT had no significant effect on its very extensive clearance with human hepatocytes, consistent with a low oxidative biotransformation and an extensive glucuronidation also observed in both human hepatic microsomal fractions in the presence of UDPGA and in recombinant human UGT1A1, UGT1A3, and UGT2B15.

This study, performed using different in vitro models (human hepatocytes, human hepatic microsomal fractions, recombinant human enzymes including CYPs, FMOs, MAOs, and UGTs) and different experimental conditions (incubation of dronedarone and its main metabolites, in the absence or presence of either enzyme cofactors or enzyme inhibitors) has clearly demonstrated that not only CYP3A4/5 but other CYP isoforms and non-CYP-dependent enzymes (M22 formation) are involved in the overall metabolic clearance of dronedarone.

In conclusion, the in vitro findings described in this study provide an understanding of the overall in vitro human hepatic metabolism of dronedarone and also describe the different enzyme systems involved in its metabolic cascade. The data described in this study are consistent with human in vivo metabolism investigation, and also with the well-described CYP3A4/5 drug interaction profile of dronedarone.

The combinational approach proposed in this study using the multiplicity of the in vitro hepatic models available today provides an in depth understanding of the metabolic behavior of a compound and improved insight on consequences of DDI.

## Abbreviations

1′-OH-MDZ: 1′-hydroxy-midazolam
ABT: 1-aminobenzotriazole
AF: atrial fibrillation
AU: arbitrary units
CYP: cytochrome P450
DDI: drug-drug interactions
DMSO: dimethyl sulfoxide
EMA: European Medicines Agency
ESI: electrospray ionization mode
fm: fraction metabolized
FMO: flavin monooxygenase
Glu-O-MDZ: midazolam-*O*-glucuronide
HEPES: 4-(2-hydroxyethyl)-1-piperazineethanesulfonic acid
HPLC-UV: high-performance liquid chromatography coupled with ultra-violet detection
LC/MS-MS: liquid chromatography/tandem mass spectrometry
MAO: monoamine oxidase
MDZ: midazolam
NADPH: nicotinamide adenine dinucleotide phosphate
NSR: normal sinus rhythm
UDPGA: uridine 5′-diphosphoglucuronic acid
UGT: UDP-glucuronosyl-transferase

## Supporting Information

**Figure S1.** Comparative ion current chromatograms obtained following a 6-h incubation of human hepatocytes (pool of four different preparations run in parallel) with 5 *µ*mol/L dronedarone either (A), alone, that is control conditions, (B) with 3 *µ*mol/L ketoconazole, (C) with 1 mmol/L 1-aminobenzotriazole, or (D) with 0.25 *µ*mol/L clorgyline.

**Figure S2.** Comparative ion current chromatograms obtained following a 6-h incubation of human hepatocytes (pool of four different preparations run in parallel) with 5 *µ*mol/L *N*-debutyl-dronedarone either (A), alone, that is control conditions, (B) with 3 *µ*mol/L ketoconazole, (C) with 1 mmol/L 1-aminobenzotriazole, or, (D) with 0.25 *µ*mol/L clorgyline.

**Figure S3.** Comparative ion current chromatograms obtained following a 6-h incubation of human hepatocytes (pool of four different preparations run in parallel) with 5 *µ*mol/L propanoic acid-dronedarone either (A), alone, that is control conditions, (B) with 3 *µ*mol/L ketoconazole, (C) with 1 mmol/L 1-aminobenzotriazole, or (D) with 0.25 *µ*mol/L clorgyline.

**Figure S4.** Comparative ion current chromatograms obtained following a 6-h incubation of human hepatocytes (pool of 4 different preparations run in parallel) with 5 *µ*mol/L phenol-dronedarone either (A), alone, that is control conditions, (B) with 3 *µ*mol/L ketoconazole, (C) with 1 mmol/L 1-aminobenzotriazole, or (D) with 0.25 *µ*mol/L clorgyline.

**Table S1.** Demographic information for cryopreserved human hepatocytes preparations. **Table S2.** Metabolic capacity of cryopreserved human hepatocytes preparations.

## References

[b1] Bourrié M, Meunier V, Berger Y, Fabre G (1996). Cytochrome P450 isoform inhibitors as a tool for the human liver microsomes. J Pharmacol Exp Ther.

[b2] Damy T, Pousset F, Caplain H, Hulot JS, Lechat P (2004). Pharmacokinetic and pharmacodynamic interactions between metoprolol and dronedarone in extensive and poor CYP2D6 metabolizers healthy subjects. Fundam Clin Pharmacol.

[b3] Daujat M, Fabre I, Diaz D, Pichard L, Fabre G, Fabre JM (1990). Inducibility and expression of class IA and IIIA cytochromes P450 in primary cultures of adult human hepatocytes. Biochem Pharmacol (Life Sci Adv).

[b4] Dorris RL (1982). A simple method for screening monoamine oxidase (MAO) inhibitory drugs for type preference. J Pharmacol Methods.

[b5] Fabre G, Rahmani R, Placidi M, Combalbert J, Covo J, Cano JP (1988). Characterization of midazolam metabolism using human hepatic microsomal fractions and hepatocytes in suspension obtained by perfusing whole human livers. Biochem Pharmacol.

[b6] Isom HC, Georgoff I (1984). Quantitative assay for albumin-producing liver cells after simian virus 40 transformation of rat hepatocytes maintained in chemically defined medium. Proc Natl Acad Sci.

[b7] Klieber S, Hugla S, Ngo R, Arabeyre-Fabre C, Meunier V, Sadoun F (2008). Contribution of the *N*-glucuronidation pathway to the overall in vitro metabolic clearance of midazolam in humans. Drug Metab Dispos.

[b8] Lawton MP, Cashman JR, Cresteil T, Dolphin CT, Elfarra AA, Hines RN (1994). A nomenclature for the mammalian flavin-containing monooxygenase gene family based on amino acid sequence identities. Arch Biochem Biophys.

[b9] Mackenzie PI, Owens IS, Burchell B, Bock KW, Bairoch A, Bélanger A (1997). The UDP glucuronosyltransferase gene family: recommended nomenclature update based on evolutionary divergence. Pharmacogenetics.

[b10] McNamara RL, Tamariz LJ, Segal JB, Bass EB (2003). Management of atrial fibrillation: review of the evidence for the role of pharmacologic therapy, electrical cardioversion, and echocardiography. Ann Intern Med.

[b11] Nelson DR, Koymans L, Kamataki T, Stegeman JJ, Feyereisen R, Waxman DJ (1996). P450 superfamily: update on new sequences, gene mapping, accession numbers and nomenclature. Pharmacogenetics.

[b12] Phillips IR, Dolphin CT, Clair P, Hadley MR, Hutt AJ, McCombie RR (1995). The molecular biology of the flavin-containing monooxygenases of man. Chem Biol Interact.

[b13] Rosamond W, Flegal K, Furie K, Go A, Greenlund K, Haase N (2008). Heart disease and stroke statistics 2008 update: a report from the American Heart Association Statistics Committee and Stroke Statistics Subcommittee. Circulation.

[b14] Schweizer PA, Becker R, Katus HA, Thomas D (2011). Dronedarone: extent evidence for its safety and efficacy in the management of atrial fibrillation. Drug Design Develop Ther.

[b15] Shimada T, Yamazaki H, Mimura M, Inui Y, Guengerich FP (1994). Interindividual variations in human liver cytochrome P-450 enzymes involved in the oxidation of drugs, carcinogens and toxic chemicals: studies with liver microsomes of 30 Japanese and 30 Caucasians. J Pharmacol Exp Ther.

[b16] Tafreshi MJ, Rowles J (2007). A review of the investigational antiarrhythmic agent dronedarone. J Cardiovasc Pharmacol Ther.

[b17] Tichy EM, Medwid AJ, Mills EA, Formica RN, Kulkarni S (2010). Significant sirolimus and dronedarone interaction in a kidney transplant recipient. Ann Pharmacother.

[b18] Touboul P, Brugada J, Capucci A, Crijns HJ, Edvardsson N, Hohnloser SH (2003). Dronedarone for prevention of atrial fibrillation: a dose-ranging study. Eur Heart J.

[b19] Wadhani N, Sarma JS, Singh BN, Radzik D, Gaud C (2006). Dose-dependent effects of oral dronedarone on the circadian variation of RR and QT intervals in healthy subjects: implications for antiarrhythmic actions. J Cardiovasc Pharmacol Ther.

[b20] Watanabe Y, Kimura J (2008). Acute inhibitory effect of dronedarone, a noniodinated benzofuran analogue of amiodarone, on Na^+^/Ca^2+^ exchange current in guinea pig cardiac ventricular myocytes. Naunyn Schmiedebergs Arch Pharmacol.

[b21] Xie C, Yang S, Zhong D, Dai X, Chen X (2011). Simultaneous determination of dronedarone and its active metabolite debutyldronedarone in human plasma by liquid chromatography-tandem mass spectrometry: application to a pharmacokinetic study. J Chromatogr B Analyt Technol Biomed Life Sci.

