# Supplementary figures and images for: Identification of metabolic pathways and enzyme systems involved in the in vitro human hepatic metabolism of dronedarone, a potent new oral antiarrhythmic drug
Source: Pharmacol Res Perspect. 2014 Apr 22;2(3):e00044. doi: 10.1002/prp2.44 (PMC4186413; doi:10.1002/prp2.44)

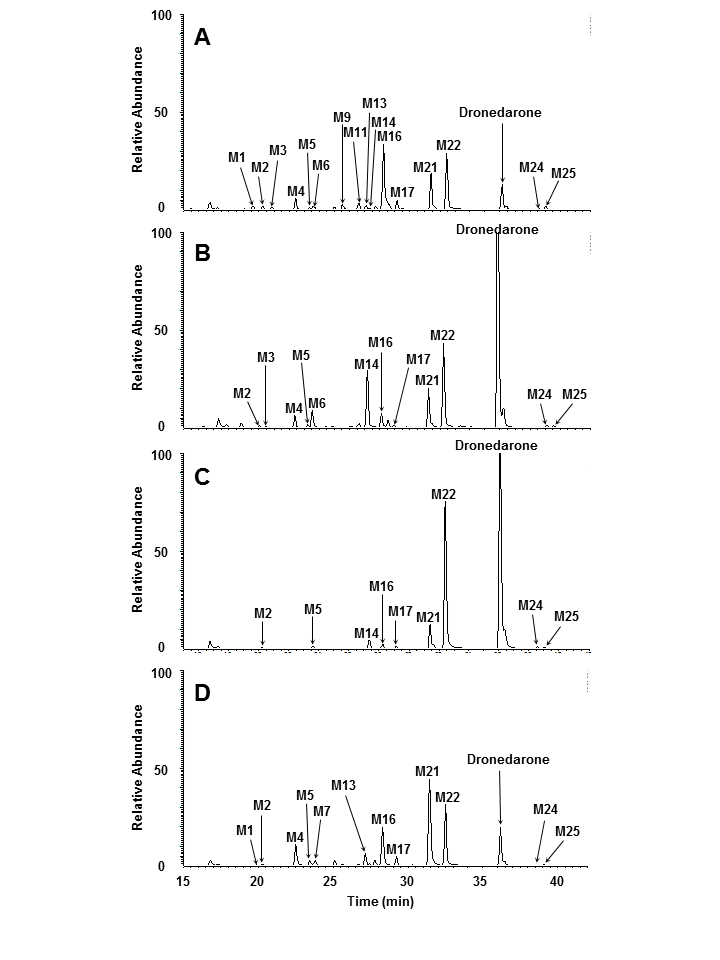

Supplement: Supplementary file 1 — Figure S1. Comparative ion current chromatograms obtained following a 6-h incubation of human hepatocytes (pool of four different preparations run in parallel) with 5 µmol/L dronedarone either (A), alone, that is control conditions, (B) with 3 µmol/L ketoconazole, (C) with 1 mmol/L 1-aminobenzotriazole, or (D) with 0.25 µmol/L clorgyline. [file prp20002-e00044-SD1.tif]

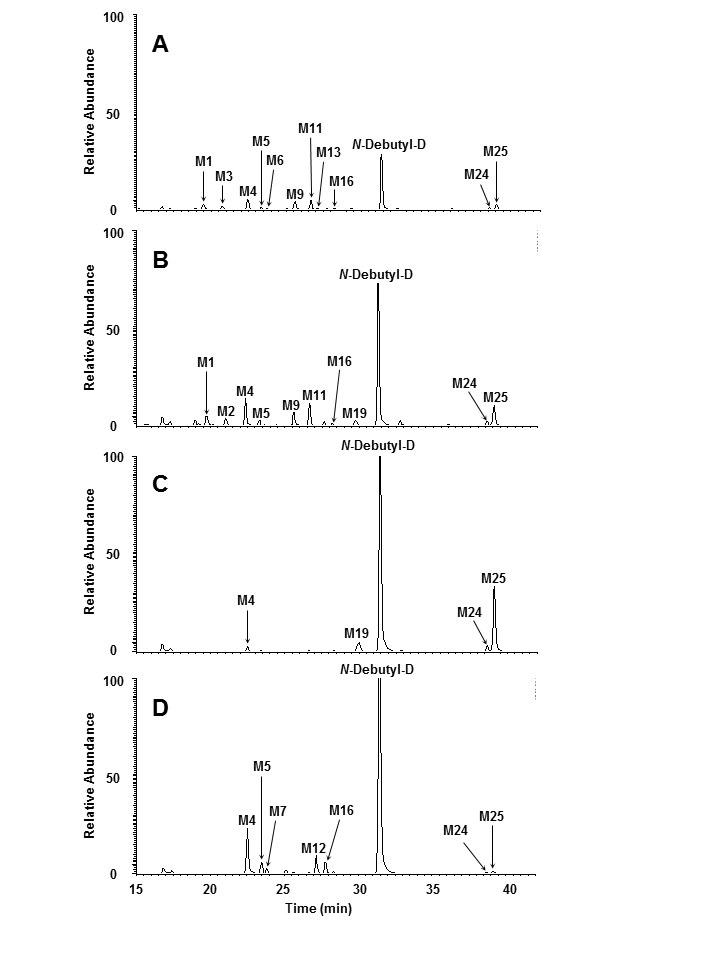

Supplement: Supplementary file 2 — Figure S2. Comparative ion current chromatograms obtained following a 6-h incubation of human hepatocytes (pool of four different preparations run in parallel) with 5 µmol/L N-debutyl-dronedarone either (A), alone, that is control conditions, (B) with 3 µmol/L ketoconazole, (C) with 1 mmol/L 1-aminobenzotriazole, or, (D) with 0.25 µmol/L clorgyline. [file prp20002-e00044-SD2.tif]

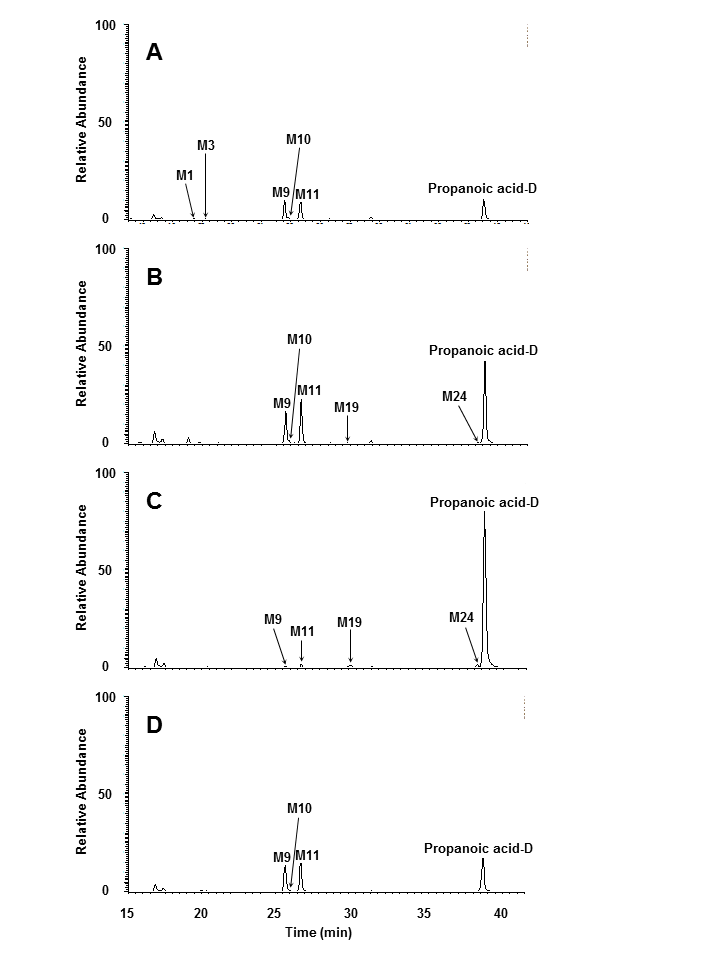

Supplement: Supplementary file 3 — Figure S3. Comparative ion current chromatograms obtained following a 6-h incubation of human hepatocytes (pool of four different preparations run in parallel) with 5 µmol/L propanoic acid-dronedarone either (A), alone, that is control conditions, (B) with 3 µmol/L ketoconazole, (C) with 1 mmol/L 1-aminobenzotriazole, or (D) with 0.25 µmol/L clorgyline. [file prp20002-e00044-SD3.tif]

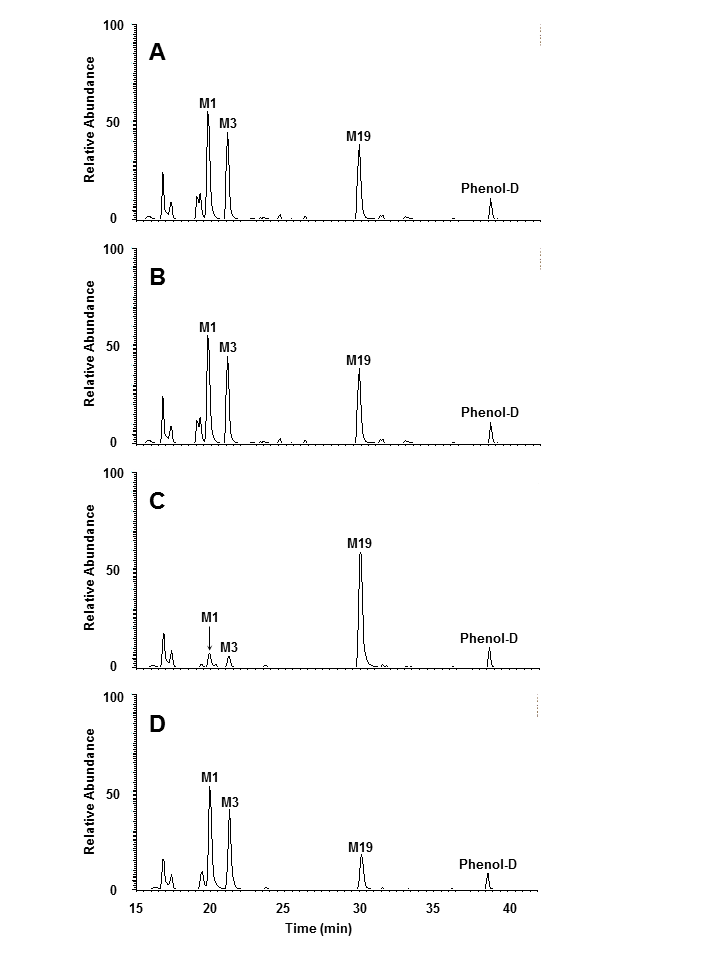

Supplement: Supplementary file 4 — Figure S4. Comparative ion current chromatograms obtained following a 6-h incubation of human hepatocytes (pool of 4 different preparations run in parallel) with 5 µmol/L phenol-dronedarone either (A), alone, that is control conditions, (B) with 3 µmol/L ketoconazole, (C) with 1 mmol/L 1-aminobenzotriazole, or (D) with 0.25 µmol/L clorgyline. [file prp20002-e00044-SD4.tif]
