# Supplementary material for: Identification of metabolic pathways and enzyme systems involved in the in vitro human hepatic metabolism of dronedarone, a potent new oral antiarrhythmic drug
Source: Pharmacol Res Perspect. 2014 Apr 22;2(3):e00044. doi: 10.1002/prp2.44 (PMC4186413; doi:10.1002/prp2.44)
Supplement: Supplementary file 5 [file prp20002-e00044-SD5.docx]

**Supplementary information**

**Supplementary Tables**

**Supplementary Table S1**: Demographic information for cryopreserved human hepatocytes preparations

| *Donor* | *Provider* | *Individual characteristics* | | | |
| --- | --- | --- | --- | --- | --- |
|  |  | *Gender* | *Age* | *Pathology* | *Ethnicity* |
| BD-91 | BD-Gentest | Male | 5 | Anoxia | Afro-American |
| BD-296 | BD-Gentest | Male | 17 | Anoxia | Caucasian |
| HU-1359 | Gibco/Lifetechnologies | Male | 58 | Not Available | Caucasian |
| IVT-IBG | Celsis/InVitroTech. | Female | 67 | Anoxia | Caucasian |

**Supplementary Table S2**: Metabolic capacity of cryopreserved human hepatocytes preparations

| *Donor* | *Viability (%)* | *Plating Efficiency* | *Metabolic capacity in nmol.h^-1^.10^-6^ cells (rating)* | | | |
| --- | --- | --- | --- | --- | --- | --- |
|  |  |  | *CYP1A2^a^* | *CYP2C9^b^* | *CYP2D6^c^* | *CYP3A4^d^* |
| BD-91 | 98 | > 95 % | 0.552 (L) | 0.019 (L) | 0.213 (L) | 1.604 (H) |
| BD-296 | 95 | > 95 % | 0.112 (L) | 0.044 (M) | 0.861 (M) | 1.531 (H) |
| HU-1359 | 97 | > 95 % | 0.228 (L) | 0.054 (M) | 0.726 (M) | 0.599 (M) |
| IVT-IBG | 99 | > 95 % | 0.171 (L) | 0.097 (H) | 0.819 (M) | 0.466 (M) |

^a^: in vitro velocity of 4-acetamido-phenol formation

^b^: in vitro velocity of hydroxymethyl-tolbutamide formation

^c^: in vitro velocity of dextrorphan formation

^d^: in vitro velocity of midazolam CYP3A4-dependent metabolites formation

Rating: L = Low, M = Moderate, H = High

**Supplementary Figures**

**Supplementary Fig. S5:** Comparative ion current chromatograms obtained following a 6-hour incubation of human hepatocytes (pool of 4 different preparations run in parallel) with 5 µM dronedarone either A), alone, i.e. control conditions, B) with 3 µM ketoconazole, C) with 1 mM 1-aminobenzotriazole, or D) with 0.25 µM clorgyline.

**Supplementary Fig. S6:**Comparative ion current chromatograms obtained following a 6-hour incubation of human hepatocytes (pool of 4 different preparations run in parallel) with 5 µM N-debutyl-dronedarone either A), alone, i.e. control conditions, B) with 3 µM ketoconazole, C) with 1 mM 1-aminobenzotriazole, or, D) with 0.25 µM clorgyline.

**Supplementary Fig. S7:** Comparative ion current chromatograms obtained following a 6-hour incubation of human hepatocytes (pool of 4 different preparations run in parallel) with 5 µM propanoic acid-dronedarone either A), alone, i.e. control conditions, B) with 3 µM ketoconazole, C) with 1 mM 1-aminobenzotriazole, or D) with 0.25 µM clorgyline.

**Supplementary Fig. S8:** Comparative ion current chromatograms obtained following a 6-hour incubation of human hepatocytes (pool of 4 different preparations run in parallel) with 5 µM phenol-dronedarone either A), alone, i.e. control conditions, B) with 3 µM ketoconazole, C) with 1 mM 1-aminobenzotriazole, or D) with 0.25 µM clorgyline.
